# Devo-Aging: Intersections Between Development and Aging

**DOI:** 10.1007/s11357-023-00809-2

**Published:** 2023-05-09

**Authors:** Carlos Giovanni Silva-García

**Affiliations:** 1 Department of Molecular Biology, Cell Biology, and Biochemistry, Brown University, Providence, RI USA; 2 Center on the Biology of Aging, Brown University, Providence, RI USA

**Keywords:** Aging, Development, Environment, Epigenetics, Age-related diseases, mTOR, IIS, FGF, TGF-b, Wnt, Hippo, Hedgehog

## Abstract

There are two fundamental questions in developmental biology. How does a single fertilized cell give rise to a whole body? and how does this body later produce progeny? Synchronization of these embryonic and postembryonic developments ensures continuity of life from one generation to the next. An enormous amount of work has been done to unravel the molecular mechanisms behind these processes, but more recently, modern developmental biology has been expanded to study development in wider contexts, including regeneration, environment, disease, and even aging. However, we have just started to understand how the mechanisms that govern development also regulate aging. This review discusses examples of signaling pathways involved in development to elucidate how their regulation influences healthspan and lifespan. Therefore, a better knowledge of developmental signaling pathways stresses the possibility of using them as innovative biomarkers and targets for aging and age-related diseases.

## Introduction

Aging is a complex, multifaceted process characterized by functional decline and increasing morbidity that eventually results in the death of an organism. Aging then induces many physiological changes accompanied by loss of homeostasis in molecular and cellular processes, affecting tissues and organs. This progressive failure during aging not only occurs in response to endogenous alterations, but environmental conditions also induce damage accumulation and physiological deterioration, resulting in susceptibility to various diseases. Throughout our life, we are exposed to several changing conditions that ultimately impact the rate of aging. A sensitive stage that significantly impacts our lives occurs during development. Therefore, research into developmental mechanisms may reveal the origins and potential treatments to prevent disease later in life.

Several theories have been proposed to explain the aging process from the molecular, cellular, organ, and organismal levels [1], and some of them link developmental processes with aging. One of the most widely cited evolutionary theories of aging, the antagonistic pleiotropy theory by Williams in 1957 [2] proposes that animals possess genes that increase fitness early in life but diminish it in later life. Those genes have multiple effects (pleiotropy); in some cases, one effect may be good for the animal while another is detrimental (antagonistic). These genes can then be favored by natural selection, even if they accelerate the aging process, because the selection is stronger early in life. Furthermore, multiple signaling pathways are activated in the early stages of life to regulate organismal growth and development. However, if these pathways remain inappropriately higher than the optimal activity (hyper-function), they may cause aging and the appearance of age-related diseases; this is the hyperfunction theory of aging proposed by Blagosklonny [3]. The developmental theory of aging proposed by Dilman suggests that mechanisms during ontogenesis provide relativity stability during the development stage but disturb homeostasis when the development and growth of the organism have ceased [4]. Another theory, the developmental origins of health and disease theory (Langley-Evans), proposes that environmental exposures during early life can permanently influence health and vulnerability to disease [5]. Considering these theories, suppose development and aging are part of the same molecular mechanisms—or at least very intersected. In that case, it is logical that adult-onset disorders, such as obesity, diabetes, cardiovascular disease, neurodegeneration, and cancer, may be linked to early life events. Therefore, life nutritional status, exposures to environmental chemicals, drugs, infections, lifestyles, or stress in parents will impact the onset of these disorders in progeny, accelerating the aging process.

The field of geroscience seeks to uncover the fundamental genetic and molecular mechanisms that drive the aging process to identify pathways and develop preventative approaches for age-related diseases. Therefore, studying developmental signaling pathways in aging (*Devo-Aging* approaches) will contribute to understanding how basic mechanisms drive the aging process. Rather than focusing on theories of development and aging—an exciting field—the purpose here is to provide insights into basic developmental mechanisms that regulate aging and lifespan determination. Consequently, this review provides an overview of the major developmental signaling pathways, explaining their molecular basis and what is known about their role in aging. The majority of signaling pathways can be reduced to a simplistic route: reception, transduction, and response (Table 1). Transmembrane receptors or other non-membrane sensors detect ligands, metabolites, or nutrients that trigger the signal cascade. Once a receptor receives a signal, it usually undergoes a conformational change, which in turn launches a series of biochemical reactions and protein–protein interactions within the cell, the transduction phase. In the end, the cell responds in a number of ways depending on the signal received, including changes in metabolism and alterations in cell shape or movement, but most converge in regulating gene expression at the transcriptional level [18]. This review examines how developmental signaling pathways responsible for generating an entire organism can regulate health and lifespan. It also discusses emerging interactions of these pathways with the environment and epigenetic modifications that ultimately regulate gene expression. Several components of these developmental signal cascades are conserved from worms to humans. Since identifying conserved gene pathways involved in regulating lifespan and life history is a central goal of aging research, studying these developmental pathways in the context of aging will therefore have direct implications for human health.

**Table 1:**
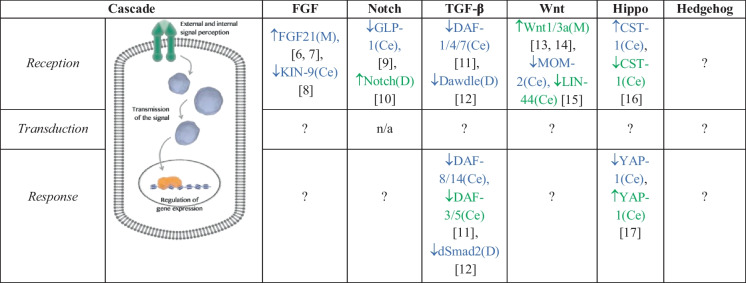
Components of understudied developmental pathways that regulate lifespan

↑, increase, active, or overexpression; ↓, mutant or knockdown; *Blue*, promote longevity; *Green*, shorten lifespan or accelerate aging; *M*, mammals; *Ce*, *C. elegans*; *D*, *Drosophila*; *n/a*, not applicable

## IIS and mTOR, master regulators of development and aging

One of the classic pathways that connect aging and development is the mechanistic target of rapamycin (mTOR). Two functionally distinct regulatory complexes involving mTOR have been identified, mTORC1 and mTORC2 [19]. The mTOR complexes regulate essential cellular processes, including ribosome biogenesis, protein synthesis, autophagy, gene transcription, mRNA turnover, vesicular trafficking, and cytoskeletal organization [20, 21]. In early mouse embryos, homozygous animals for *mTOR* deletion die at around 7 days-post-coitum due to impaired cell proliferation and gastrulation [22]. The mTOR gene is also necessary for the normal growth of worms and flies [23–25]. However, the downregulation, mainly in adulthood, of mTOR complex components extends lifespan across different species [26–32]. Hence, it is exciting to understand how mTOR signaling differs in developmental and post-developmental periods in metazoans.

Inhibition of the mTORC1 pathway through genetic depletion of mTOR or the regulatory-associated protein of mTOR (raptor) has been shown to extend lifespan in a variety of species, including yeast, worms, flies, and mammals [29, 31–34]. In addition to genetic approaches, lifespan extension can also be achieved by inhibiting mTOR regulators or mTOR targets as well as by pharmacological interventions. Since mTOR complexes modulate cellular signals in response to environmental changes in insulin/growth factors, amino acids, energy, and oxygen, many mTOR upstream and downstream components have been described [35]. However, we know less about how exactly these mTOR components participate in the biology of aging. For instance, nutrients regulate mTORC1 by activating the Rag GTPases RagaA and RagaC, which recruit mTORC1 to the lysosomal surface, an essential step for its kinase activation by growth factors such as the insulin/IGF1-PI3K-Akt signaling and the ERK1/2 MAPK cascade [36–41]. Although RagA is indispensable for embryonic development in mice [42], *C. elegans* with a null mutation *raga-1* (ortholog of RagA) are long-lived via maintaining mitochondrial fusion [43, 44]. Although null *raga-1* worms have impaired growth and reproduction [43, 44], recent work shows that neuronal degradation of RAGA-1 does not impair growth, slow development, or decrease reproductive capacity but extends lifespan [45]. Dietary interventions have been extensively studied as extenders of lifespan and delayers of age-associated pathologies [46]. Therefore, upstream regulators of the mTOR signaling pathway need special attention here to study mechanisms of nutrient sensing, which will provide important insights into biological aging. mTOR targets have also been associated with aging and longevity. Inactivation of the ribosomal S6 kinase 1 (S6K1), a downstream target of mTORC1, leads to increased lifespan in yeast, worms, flies, and mammals [29, 30, 43, 47]. Although the molecular mechanism that governs this S6K1-dependent phenotype is not well defined, perhaps S6K1 extends lifespan by reducing the production of misfolded proteins due to its role in translation.

Pioneering work shows that pharmacological inhibition of mTOR signaling by methionine sulfoximine or rapamycin increases yeast chronological lifespan [34]. After this discovery, other researchers around the world have shown that rapamycin also extends the lifespan in mice, *Drosophila*, and *C. elegans* [48–50]. Initially, it was demonstrated that rapamycin increases mouse lifespan when administered late in life [48], but recent evidence indicates that administration of rapamycin during development is sufficient to extend lifespan and healthspan in mice and even in other distal species such as the planktonic crustacean *Daphnia magna* and *Drosophila* [51, 52]. These data suggest that early-life rapamycin treatment operates through evolutionarily conserved longevity mechanisms and opens the door to exploring how pharmacological interventions during a specific time window in early life or development affect later life of the animal, especially susceptibility to diseases associated with age. Due to the broad potential of the mTOR, studying this signaling pathway in metabolism, cancer, and aging has been attractive to many research groups recently [26, 53–55]. However, mTOR signaling plays an essential role during embryonic development by regulating growth and proliferation [56, 57]. Although the data indicate that the mTOR signaling controls aging through conserved evolutionary mechanisms, regulation of embryonic development should substantially differ between species. For instance, embryonic cell divisions occur in the complete absence of growth in *C. elegans* [58]. Therefore, understanding subtle variations of embryonic mTOR complexes will help to discover new unidentified components and regulators in the pathway, which could be targeted in a wide range of postembryonic mTOR-related disorders and aging.

Along with the mTOR pathway, insulin and insulin-like growth factor (IGF) signaling (IIS) is a master regulator of growth, metabolism, and aging. The IIS system is a complex network of ligands, receptors, and signaling pathways. IIS activates mTOR via class I phosphatidylinositol 3-kinase (PI3K)/Akt, promoting cell growth by inducing protein, lipid, and nucleotide synthesis and inhibiting autophagy [54, 55, 59]. The IIS pathway is considered to be important in growth and development in addition to its central role in metabolic homeostasis. Mice mutants in the IGF type 1 receptor (*Igf1r*) die at birth [60], and loss of function mutations in the *inr* gene in *Drosophila* also lead to embryonic lethality [61]. However, *C. elegans* mutants in the *daf-2* gene (homolog of IGF-1R) pass the embryo stage but show a larval arrest phenotype [62]. These data suggest a difference in metabolic contribution during development in different organisms. Metabolism is regulated across various spatial and temporal scales. Thus, parental metabolic perturbations that damage several tissues, such as blood vessels, nerves, muscles, and physiology in general, will have repercussions on the embryo’s development and can be responsible for future metabolic disorders, especially in those species like us with gestation. Although IIS is critical to maintaining homeostasis and preventing metabolic diseases in adulthood, this pathway has been the keystone of the aging field. The discovery of genetic manipulation of aging by mutations in two components in the IIS pathway in *C. elegans*, *age-1* (orthologue of PI3K) and *daf-2* [63, 64], led to tremendous work to test conservation in other organisms [60, 65–69]. However, we have just started to define its spatiotemporal contribution to longevity. In *C. elegans*, intestine-specific removal or end-of-life targeted degradation of DAF-2 doubles the lifespan [70, 71]. These data indicate that when and where the IIS signaling pathway is regulated determines its contribution to aging and point out to define the spatiotemporal contribution of other developmental pathways in aging.

In addition to mTOR, the IIS pathway is highly linked to the growth hormone (GH) signaling pathway in mammals, promoting postnatal longitudinal growth. GH stimulates IGF-1 production, and the GH pathway regulates lipid, carbohydrate, nitrogen, and mineral metabolism. It increases lipolysis in adipocytes, decreasing body fat; it increases amino acid uptake and nitrogen retention in muscles and maintains muscle mass and strength; it also impacts multiple systems such as cardiovascular, immune, and central nervous systems [72]. Consequently, abnormal GH secretion has the potential to impact multiple tissues and organs. Decreased GH-IGF-1 signaling has been shown to extend longevity in mice [73]. Dwarf mice are GH-deficient animals, and three dwarf mice lines (Ames, Snell, and Laron) have an extended lifespan with reduced weight and reduced levels of IGF-1, insulin, and glucose [74]. With a dramatic lifespan extension of around 60% [75], the Ames dwarf mice have been subjected to different studies to unravel the mechanisms that favor this phenotype. But more recently, Sun et al. show that an early-life GH treatment (a week old) in Ames dwarf mice can have a long-lasting effect on the animals’ lifespan [76]. These Ames dwarf animals subject to early-life GH treatment grew longer and heavier and with a significantly shorter lifespan by activation of proinflammatory pathways (including JNK and NF-kB) and suppression of xenobiotic signals in the liver [76]. These data exemplify how events early in life have a lasting effect on aging and lifespan. Therefore, identifying specific developmental windows is critical to understand how these early life events affect the risk of developing age-related diseases.

## Developmental pathways understudied in aging

### FGF

Fibroblast growth factors (FGF) and their receptors (FGFR) serve many functions in both developing and adult organisms. FGFs are broad-spectrum mitogens and are expressed in nearly all tissues. They regulate organogenesis in the earliest stages of embryonic development, and in the adult, they function as homeostatic factors that are important for tissue maintenance, repair, regeneration, and metabolism. Dysregulation of FGF signaling is associated with a variety of human diseases, including congenital craniosynostosis, dwarfism syndromes, chronic kidney disease, obesity, insulin resistance, and cancer [77]. Mice and humans have twenty-two FGF ligands and four FGFRs, while in invertebrates these numbers go down to three FGF ligands and two FGFRs in *Drosophila*, and two FGF ligands and a single FGFR in *C. elegans* [77–81]. Although these differences in the number of ligands and receptors between species show how this pathway has changed during evolution, the basic mechanism of activation is conserved. The binding of FGFs to the inactive monomeric FGFRs triggers conformational changes in FGFRs, resulting activation of downstream signaling molecules. The classical FGF/FGFR downstream signals include Ras/Raf-MEK-MAPKs (mitogen-activated protein kinases), PI3K/AKT, PLCγ, and signal transducer and activator of transcription (STAT) [82]. During embryonic development, FGF/FGFR signaling regulates organogenesis, cell migration, morphogenesis, and neuronal induction and patterning [83]. More recent studies are focusing on the role of this pathway in age-related metabolic diseases and aging [84]. In particular, FGF 21 whose increase favors oxidation of free fatty acids and inhibits lipogenesis in the liver to supply energy when glucose levels are low or caloric restricted [84]. FGF21 is induced by fasting, and transgenic overexpression of FGF21 markedly extends lifespan in mice [6, 7]. Supporting the conserved role of the FGF/FGFR signaling in aging, the silkworm *Bombyx mori* growth with an FGF21 replenishment showed a significant increase in lifespan [85]. In addition, the absence of the *C. elegans* FGFR4 homolog KIN-9 promotes longevity [8]. These recent data highlight a novel role of FGF/FGFR signaling in aging. However, the molecular mechanism by which this signaling induces longevity is not yet defined. Since FGF/FGFR signals are activated during a low energy state like caloric restriction, one possibility is that this pathway promotes longevity through modulation of energetic metabolism.

### Notch

Notch signaling is an evolutionarily conserved cell signaling that displays pleiotropic functions in almost every tissue. It involves several developmental cell-fate decisions, including neuronal development, angiogenesis, vasculogenesis, cardiac development, and maintenance of neural stem cells [86–92]. Due to its importance in development and tissue function, disruption of the Notch signaling leads to several diseases, including cardiac and skeletal disorders, and it is associated with syndromes such as Alagille and Hajdu-Cheney [93, 94]. The Notch pathway has a simple linear signaling axis, and unlike other signaling pathways that are amplified via kinase cascades, this pathway does not contain any intermediate that amplifies the signal. This signal is activated by the interaction between five Notch ligands encoded by JAG1, JAG2, DLL1, DLL3, and DLL4, and four transmembrane receptors encoded by Notch genes, NOTCH1-4 [95]. When the cell-surface receptor Notch interacts with a ligand, its Notch intracellular domain (NICD) is cleaved and then translocates to the nucleus to regulate transcriptional complexes containing the DNA-binding protein CBF1/RBPjk/Su(H)/Lag1 (CSL) [96]. CSL is a DNA-binding adaptor, and canonically, CSL binds NICD in the nucleus. This interaction recruits several proteins to activate transcription, including Mastermind-like (MAML) adaptor protein, histone acetyltransferases such as p300, and other transcriptional components [95]. This complex then drives the expression of downstream target genes required for proper development [97, 98]. Originally, this pathway was discovered in *Drosophila* and *C. elegans*, which have one and two Notch receptors, respectively [95]. Like most developmental pathways, disruption of the Notch signaling leads to lethal effects. However, manipulation of specific steps in the cascade contributes to a lifespan benefit. A classic example is the *C. elegans glp-1* mutant [9]. The longevity in *glp-1* mutants has been attributed to the absence of the germline and not a specific function of the *glp-1* gene [9]. However, other germline-less mutants (i.e., *glp-4*) do not show a lifespan extension [99]. Although other conditions, such as germline ablation, increase lifespan in *C. elegans* [100], the mechanism that modulates GLP-1-dependent longevity should be related to the regulation of Notch signals. Supporting this notion, an active form of Notch, specifically in intestinal stem cells in *Drosophila*, shortens lifespan [10]. In addition, a growing number of recent studies highlight the contribution of Notch signaling in various pathological processes associated with age, including cancer, cardiovascular and metabolic diseases, and Alzheimer’s [101–103]. Therefore, studies focusing on the temporal regulation of components in the Notch pathway, particularly in adult life, where it is essential in maintaining tissue-specific homeostasis, will elucidate new mechanisms that modulate aging and the onset of age-related disorders.

### TGF-β

The transforming growth factor-β (TGF-β) is a superfamily of evolutionarily conserved cytokines that regulates diverse cellular activities, such as growth, adhesion, migration, and differentiation in embryonic development. In mammals, this family has over thirty members, including TGF-βs, activins, bone morphogenetic proteins (BMPs), and growth differentiation factors (GDFs) [104, 105], and many orthologs are known in *Drosophila* and *C. elegans* [106, 107]. Due to the notable role of TGF-β signaling in cell proliferation and survival, this signaling pathway is involved in multiple aspects of cancer biology [108], providing important possibilities as an effective therapeutic target. The family is divided into two general branches: the BMP/GDF and TGFβ/activin/nodal. Signaling is initiated with ligand binding to two related serine-threonine kinase transmembrane receptors (type I and II). This activated ligand/receptor complex then recruits and phosphorylates the intracellular mediators (Smads), which form complexes with each other and other proteins to modulate transcription of target genes in the nucleus [109]. There, the activated Smad complex associates with two classes of proteins: DNA-binding cofactors that help select target genes and coactivators or corepressors that determine the transcriptional rate. Dysregulation of this cascade is associated with several age-related disorders. Upregulation of TGF-β signaling is detected in the brain of aged individuals and during degenerative conditions such as osteoarthritis [110, 111]. Therefore, similar to the mTOR pathway, the TGF-β pathway is critical in keeping healthy tissues during development and in young individuals; however, this pathway starts to promote damage as we age. Although no evidence directly links TGF-β signaling with regulation of lifespan in mammals, it has been shown that this family regulates longevity in *C. elegans* when animals skip developmental defects. *C. elegans* TGF-β mutants show an egg-laying defect, in which delayed egg-laying results in shortened life span because embryos hatch inside the mother leading to matricide. When animals are exposed to the DNA synthesis inhibitor FUdR, which blocks embryonic development, they show a longevity phenotype [11]. Interestingly, in *Drosophila*, reduced activity of the activin ligand Dawdle or its downstream Smad, dSmad2, also leads to an increased lifespan [12]. Studying the age-related molecular mechanisms of developmental pathways in model organisms will elucidate insights into human aging. For instance, several potential anti-TGF-β inhibitors [112, 113] can be tested in these model systems to assess their role and life-long health consequences.

### Wnt

The Wnt signaling pathway is an ancient and evolutionary conserved pathway that regulates a wide range of cellular functions during development and adulthood. In development, the Wnt pathway regulates embryonic patterning and morphogenesis by modulating cell proliferation, cell fate determination, apoptosis, cell migration, and cell polarity. As a result, mutations in the Wnt pathway are often linked to human congenital disabilities, cancer, and other diseases in adults [114]. The canonical Wnt pathway is activated by binding extracellular Wnt ligands (including Wnt3a, Wnt1, and Wnt5a) to membrane receptors (Frizzled and LRP5/6) by autocrine/paracrine signals. Once activated, the Wnt pathway induces the stability of β-catenin, which then translocates to the nucleus, serving as a coactivator for TCF to active Wnt-responsive genes. Without Wnt ligands, cytoplasmic β-catenin forms a complex with Axin, APC, GSK3, and CK1 and is phosphorylated by CK1 and GSK3. This phosphorylation targets β-catenin to proteasome degradation via the E3 ubiquitin ligase β-Trcp [115, 116]. Although regulation of Wnt signaling is critical for many developmental cellular functions, its role in aging is contradictory in different organisms. Activating Wnt signaling in mammals inhibits amyloid-β production and tau protein hyperphosphorylation in the Alzheimer’s disease (AD) brain [117]. Furthermore, loss of Wnt signaling induces AD-like pathology in an AD mouse model [118]. In contrast, other works suggest that Wnt signaling accelerates the onset of aging. Circulating Wnt ligands Wnt3a and Wnt1 in the bloodstream of older mice promotes muscle fibroblasts and accelerated cellular senescence [13, 14]. The Wnt pathway also has dual roles in *C. elegans* aging. Of the five genes that encode Wnt ligands in worms, the knockdown of three (*egl-20*, *cwn-1*, and *cwn-2*) does not affect longevity, while *mom-2* increases lifespan, and *lin-44* shortens lifespan [15]. These results imply that, like in mammals, in *C. elegans* the Wnt signaling plays different roles in aging. Therefore, more work is needed to dissect its components in aging by determining their levels and site of endogenous expressions throughout the entire lifespan.

### Hippo

The Hippo pathway activity controls the dynamic localization of the transcriptional regulators YAP and TAZ between nucleus and cytoplasm. When the Hippo pathway is off, YAP/TAZ are dephosphorylated and accumulate in the nucleus, where they can bind various transcription factors, most notably the TEAD family. When the Hippo pathway is on, YAP and TAZ activity is regulated by the LATS1 and LATS2 kinases, which phosphorylate YAP/TAZ on conserved residues, resulting in their binding to 14–3–3 proteins and cytoplasmic retention as well as proteasome degradation. Various upstream effectors of the LATS1 and LATS2 kinases have been identified, including the MST, MAP4K, and TAOK families of kinases, which phosphorylate and activate LATS1/2 [119, 120]. The Hippo pathway is an evolutionarily conserved signaling pathway with essential roles in organ development, stem cell homeostasis, tissue regeneration, wound healing, and immune modulation. Although dysregulated YAP/TAZ-TEAD activity is associated with various diseases in mammals, most notably cancer [121], the evidence of its role in aging comes from another model organism. *C. elegans* mutants in the *yap-1*/YAP gene exhibit enhanced lifespan, while exogenous YAP-1 expression causes a short lifespan [17]. In contrast, overexpression CST-1 (ortholog of MST in worms) extends lifespan in a DAF-16/FOXO-dependent manner, and its inactivation shortens lifespan [16]. In support of the Hippo pathway role in age-related mechanisms, work in mice shows that impaired liver regeneration in aged animals can be rescued by silencing Hippo kinases MST1 and MST2 [122]. These data suggest different roles of the Hippo pathway in aging. Therefore, more work on this pathway in model organisms is needed to discover its potential in preventing or treating age-related diseases. The Hippo pathway has also been linked to other longevity regulators [123]. For example, the AMP-activated protein kinase (AMPK), which is an ancestral energy sensor and key to signaling the promotion of healthy aging and longevity [124, 125], phosphorylates YAP on multiple sites and inhibits its transcriptional activity [126, 127]. But it is undetermined whether YAP/TAZ-TEAD activity downstream of AMPK is required to promote AMPK-dependent longevity. The Hippo pathway has been associated with other longevity pathways, such as sirtuins and autophagy [123], though these longevity mechanisms are not defined.

### Hedgehog

The Hedgehog (Hh) family of secreted proteins is used during embryonic development for intercellular communication. Hh is essential for growth, patterning, and morphogenesis in invertebrates and mammals. Since the discovery of the Hh gene in *Drosophila*, *hh* [128], much progress has been made in revealing its molecular cascade and role in development and disease [129]. Hh is a morphogen secreted by one cell and then sensed by a different cell. This will activate a cascade signaling that ends in the activation of gene expression. There are three mammalian Hh proteins, Shh, Indian-Hedgehog (Ihh), and Desert-Hedgehog (Dhh) [130]. The activation of this pathway occurs when Hh binds the 12-transmembrane protein Patched (Ptch1). In response to this binding, Ptch no longer inhibits Smo, which initiates the downstream signaling pathway cascade by activating the transcription factors Gli [131]. Not surprisingly, malfunction of Hh signaling contributes to numerous human disorders, including birth defects, such as Gorlin syndrome and Greg cephalopolysyndactyly syndrome [132]. Although Hh signaling is an active pathway during embryogenesis, it seems to be silenced in adults [133]. Conversely, the downregulation of Hh pathway is associated with age-related diseases such as type 2 diabetes, neurodegeneration, atherosclerosis, osteoporosis, and cancer [134, 135]. This association with age-related disorders suggests that activation of Hh in adults can potentially enhance lifespan. However, there is no evidence demonstrating this hypothesis. One possibility by which the Hh signaling could regulate lifespan is by maintaining healthy cellular communication. Hh pathway communicates cells during development; therefore, a basic understanding of mechanisms of cellular communication will open the door to elucidating how cells and tissues lose communication with age.

## Emerging interactions: environment and epigenetics

It is not questionable that environmental exposures during pregnancy profoundly impact the developing embryo. Remarkable efforts have been made to identify chemicals that impair human fetal growth. Several epidemiologic studies link environmental pollutants to perturbations in fetal growth and development [136–138]. Maternal stress also impacts the developing embryo. For example, elevated levels of the stress hormone cortisol in the mother negatively affect offspring cognition, health, and educational attainment [139]. Social stress during pregnancy results in offspring with low birth weight, a risk factor for multiple adulthood diseases [140]. In addition to maternal contributions, early-life nutrition can have a long-term effect on the onset of diseases in humans, rodents, and other model organisms [141–143]. For instance, dietary yeast restriction during *Drosophila* development induces long-term changes in adult triglyceride storage, xenobiotic resistance, and lifespan [144]. Although the effects of environmental hazards and other conditions on the developing human embryo are very well documented, we know much less about the consequences for the forthcoming adult and future generations, as well as the molecular mechanisms that govern these processes. Therefore, studies in model organisms will help to understand how gene expression is modulated due to environmental conditions and provides the basis for developing future therapies.

Epigenetics serves as a link between the environment and gene expression. Recent studies have shown that epigenetic modifications are responsible for changes in energy metabolism, behavioral state, and longevity when animals are exposed to different environmental conditions. Furthermore, subsequent generations can inherit these changes [145–151]. On the other hand, most—if not all—developmental signaling pathways ultimately regulate gene expression by modulating transcription. Then, their interaction and regulation of chromatin modifiers are crucial in maintaining homeostasis during development. Therefore, modifications in developmental signals will impact the epigenetic landscape, potentially determining health and lifespan in the future adult. Recent works highlight the connection between developmental pathways and epigenome. Work in *C. elegans* indicates that developmental signaling plays a role in epigenetic inheritance. The TGF-β ligand DAF-1 promotes avoidance of pathogenic bacteria, and it is required for transmission of the learned behavior [149, 152]. In mammals, R-SMAD, a central transcription factor of TGF-β signaling, can recruit various epigenetic regulators (including SWI/SNF complex and histone de/acetyltransferases) to shape the transcriptome [153]. Additionally, studies in *Drosophila* and mammals have shown a role for TGF-β in neuronal plasticity that governs learning and memory processes [154]. However, whether these interactions are required to inherit non-genetic animal traits in these two species is unknown.

Other developmental signaling pathways are linked to chromatin modifications, although they are still understudied. For example, recent work shows that Wnt signaling preserves mouse embryonic stem cells (mESCs) identity and genome stability by regulating DNA methylation levels [155]. Notch activation causes a loss of H3 trimethylation (H3K27me3), a repressive chromatin mark [156]. This pathway also interacts with the histone demethylases KDM5A and KDMI/LSDI, which regulate H3K4me3 [157, 158]. These discoveries strengthen the link connecting the Notch signaling to histone modifications. But other chromatin-modifying proteins are also identified as interactors of Notch, including the nucleosome remodeling complex SWI/SNF [159]. Another developmental pathway that interacts with the SWI/SNF complex is the Hippo signaling. The Hippo-SWI/SNF interaction, along with the chromatin protein GAGA factor, regulates linage specification in mammals and cell proliferation in *Drosophila* [160]. Components of the Hippo cascade are associated with other chromatin changes. In *Drosophila*, the single YAP/TAZ homolog, Yorkie (Yki), interacts with Ncoa6, a subunit of the Trithorax-related H3K4 methyltransferase complex. Ncoa6 functions as a positive regulator of the Hippo pathway by regulating H3K4 methylation at Hippo targets genes for normal tissue growth [161]. These findings not only reveal the importance of histone modifications in controlling tissue growth but the role of developmental signals in these epigenetic processes. Although the role of developmental signaling pathways in the epigenetic regulation of gene expression is growing, more work is needed to understand how environmental conditions influence these pathways and chromatin changes, as well as the repercussions for lifespan.

The relationship between epigenetic modifications and developmental pathways is not exclusively related to the activation of gene expression. Developmental pathways also silence genes through chromatin remodeling. Brg, a chromatin remodeling factor, is required to repress Hh (Hedgehog)-dependent target genes by interacting with the Hh transducer transcription factor Gli3 in neuronal progenitors and fibroblasts [162]. Hh signaling also induces an epigenetic switch to activate gene expression. Stimulation of Hh signals recruits the demethylase Jmjd3 to remove the repressive mark H3K27me3 at genes required for proper animal development [163]. These data underline the dual functions of developmental pathways in chromatin modifications, and since these pathways are critical for gene expression during the embryogenesis of all animals, it is reasonable that they play an essential role in the establishment of epigenetic changes, which in turn could impact healthy aging.

## Concluding Remarks

Proper regulation of developmental processes is critical for the formation of tissues and organs in the embryo, and their dysregulation is fatal for the developing fetus. Developmental pathways also play a role in post-developmental processes, and perturbation in these signals is associated with age-related diseases, such as cancer, metabolic disorders, and neurodegenerations. Therefore, the mechanisms that modulate developmental pathways have the potential to have a causal role in healthy aging. Indeed, this review summarizes the work done to analyze the effects of modulating these developmental pathways in aging and longevity. Since these developmental pathways are broadly required for embryo survival, it is important to define precisely when and where during development (or post-development) each of these pathways acts to modulate aging. However, we should remember that these pathways are not isolated (as anything in the cell), and they crosstalk during development and disease [164–166]. Thus, some positive or negative effects on aging could be masked by interactions with other developmental pathways or other fundamental cellular processes required for proper development and healthspan, like autophagy [167, 168]. As a consequence, not all components in a specific pathway will have the same outcomes for lifespan (Table 1). Finally, we have a vast knowledge of molecular mechanisms during development [169], but the era of molecular aging is just beginning. We should then take advantage of this knowledge and use a multifaceted Devo-Aging approach to explore and identify new mechanisms that drive the aging process.
